# Prevalence, predictors and outcomes of bleeding events in patients with COVID-19 infection on anticoagulation: Retrospective cohort study

**DOI:** 10.1016/j.amsu.2021.102567

**Published:** 2021-07-21

**Authors:** Ahmed Alkhamis, Yousef Alshamali, Khaled Alyaqout, Eisa lari, Moh A. Alkhamis, Saad Althuwaini, Ali Lari, Maryam Alfili, Ali Alkhayat, Mohammad H. Jamal, Salman Alsabah

**Affiliations:** a Department of Surgery, Faculty of Medicine, Health Sciences Center, Kuwait University, Kuwait; b Department of Surgery, Jaber Al-Ahmad Hospital, Kuwait; c Department of Medicine, Jaber Al-Ahmad Hospital, Kuwait; d Department of Epidemiology and Biostatistics, Faculty of Public Health, Health Sciences Center, Kuwait University, Kuwait; e Orthopedic Surgery Department, Al Razi Hospital, Kuwait; f Kuwait Ministry of Health, Kuwait

**Keywords:** COVID-19, Bleeding, Anticoagulation, Dose, Mortality

## Abstract

**Background:**

This study aims to examine risk factors and complications associated with bleeding events in patients with COVID-19 who are on anticoagulation.

**Material and methods:**

We conducted retrospective review of all patients who were admitted with COVID-19 and developed bleeding events between March and June 2020. Data were analyzed in accordance with three major outcomes. Mortality within 30 days of bleeding episode, resolution of the bleeding event, and the type of bleeding event.

**Results:**

Of 122 bleeds, there was 55 (28 %) gastrointestinal (GI) bleeds. Overall mortality was 59 % (n = 72). The prevalence of therapeutic invasive interventions was 11.5 % (n = 14) all were successful in resolving the bleeding event. We found that having a GI bleeds was associated with higher risk of mortality compared to non-GI bleeds (p = 0.04) and having occult bleeds to be associated with 15 times increased risk of mortality (OR 15, 95%CI 1.97–29.1, p = 0.01). Furthermore, patients who were on no anticoagulation (none) (OR 0.1, 95%CI 0.01–0.86, p < 0.00), on prophylactic dose anticoagulation (OR 0.07, 95%CI 0.02–0.28, p = 0.03) or intermediate dose anticoagulation (OR 0.36, 95%CI 0.09–1.34, p = 0.13) were less likely to die than patients on therapeutic dose.

**Conclusions:**

The best approach to manage COVID-19 bleeding patients is to prioritize therapies that manage sepsis induce coagulopathy and shock over other approaches. In COVID-19 patients’ routine prescription of supra-prophylactic dose anticoagulation should be revisited and more individualized approach to prescription should be the norm. Regardless of the cause of bleeding event it appears that the majority of bleeding events resolve with noninvasive interventions and when invasive interventions were necessary, they were associated with high success rate despite the delay.

## Highlights

• The use of anticoagulation increases the risk of mortality.
• The routine use of supra-prophylactic dose anticoagulation should be revisited.
• Most bleeding events do resolve without surgical intervention.
• The main treatment focus should be to control sepsis induced coagulopathy.

## List of abbreviations

COVID-19: Coronavirus disease 19
GI: gastrointestinal
DIC: disseminated intravascular coagulation
ISTH: the international society of thrombosis and hemostasis
LMWH: low molecular weight heparin
UFH: unfractionated heparin
WHO: World Health Organization
PCR: polymerase chain reaction
ARDS: Acute respiratory distress syndrome
qSOFA: quick sequential organ failure assessment score
CCI: charlson comorbidity index
NPA: nasopharyngeal
APC: argon positron coagulation
IQR: interquartile ranges
EGD: esophagogastroduodenoscopy
PPI: proton pump inhibitors
ICU: intensive care unit
COPD: chronic obstructive pulmonary disease
GCS: Glasgow coma scale
WBC: white blood cells
INR: international normalized ratio
eGFR: e-glomerular filtration rate
CRP: C reactive protein
PCT: Procalcitonin
OR: odds ratio

## 1 Background

The rapid emergence of the novel coronavirus 19 (COVID-19) has brought the world to a standstill. The transmissibility and associated morbidity and mortality of this virus have overwhelmed many worldwide healthcare systems, resulting in an urgent need to understand this virus and its associated effects better.

It appears that the principal cause of death is acute respiratory failure complicated by a concomitant coagulation disorder that can induce disseminated intravascular coagulation (DIC) [1]. In light of this, anticoagulation therapy has been introduced recently as an adjuvant treatment, showing promising results in term of reducing mortality rate in several small retrospective studies [2]. As a result, many organizations including the international society of thrombosis and hemostasis (ISTH) are recommending specific anticoagulation regimens for COVID-19 patients [3]. Recommendations included the use of low molecular weight heparin (LMWH) at various doses or unfractionated heparin (UFH) infusions in COVID-19 patients with elevated D-dimer levels but no known thrombotic complications [4,5]. However, others have argued against empiric escalation of anticoagulation due to fears of a potential still unquantified increased risk of bleeding [6]. Furthermore, COVID-19 induced thrombocytopenia and DIC has been hypothesis to contribute to further increased risk of bleeding as a direct or a sepsis induced effect [7].

Because of these changes in practice, we predicted an increase in inpatients’ consults to manage patients with bleeding and hence it was imperative to appropriately identify high risk patients for bleeding so that we can mitigate potential bleeding episodes and the associated morbidities and mortality. To date there is a lack in studies evaluating risk factors associated with increased risk of gastrointestinal (GI) and other bleeding events, as well as factors associated with the resolution of bleeding episodes after interventions and the risk of mortality following bleeding events in patients with COVID-19 on anticoagulation. Our study aims to investigate these using large sample size.

## 2 Methods

The study was approved by Kuwait Ministry of Health Ethical Review Board (Ethical approval number. 1402/2020). All patients admitted to Jaber Al-Ahmad Al-Sabah hospital in Kuwait, with a diagnosis of COVID-19, based on the World Health Organization (WHO) interim guidance [8] and have been confirmed by laboratory testing using polymerase chain reaction (PCR) testing, between March 2020 and June 2020 were included. Patients who had equivocal PCR tests were excluded from the study.

### 2.1 Data collection

Data regarding patients’ demographics, baseline characteristics, inpatient therapies, complications, at time of consultations symptoms, laboratory values and interventions were collected retrospectively from the hospital electronic medical record system. These data were entered by the admitting resident prospectively.

### 2.2 Definitions

With regard to the anticoagulation dose, this variable was divided to either none, prophylactic, intermediate or therapeutic dose. Intermediate dose was defined as a dose which was higher than the criteria for prophylactic dose and lower than the one for therapeutic. The intermediate dose was adjusted according to the patient's comorbidities, laboratory values such as D-dimers, and the risk of bleeding as deemed by the treating physician. All patients diagnosed with COVID-19 stayed in the hospital until they had resolution of symptoms; deﬁned as being afebrile for more than 72 h and having oxygen saturations equal to or above 94 %. Discharge occurred after two consecutive negative PCR tests for COVID-19, more than 24 h apart. Interventions were defined as invasive, noninvasive or hemostatic. Noninvasive intervention was defined as withholding anticoagulation/antiplatelet therapies, reducing the dose of anticoagulation, transfusion of blood products, nasal packing, bladder continuous irrigation, and or instigating medications such as proton pump inhibitors and vitamin K. Invasive interventions included upper endoscopy (gastroscopy, laryngoscopy and bronchoscopy), lower GI endoscopy and traditional angiography. Hemostatic interventions included a surgical operation to control the bleeding, angioembolization, use of gold probe, epinephrine injection, argon positron coagulation (APC) and hemoclips to control an active GI bleed. Patients' mortality was tracked up to 30 days after bleeding event consultation.

### 2.3 Outcome measured

Data were analyzed in accordance with three major outcomes. First was mortality within 30 days of bleeding episode. Second was resolution of the bleeding event after consultation. Third was bleeding event outcome which was defined as either developing a GI or non-GI bleeding event. Gastrointestinal (GI) bleeding event outcome included bleeding from the upper or lower GI systems. Non-GI bleeding event outcome included all bleeding events other than GI bleeds such as abdominal wall hematoma, retroperitoneal, intraperitoneal, genitourinary, nasopharyngeal (NPA), central nervous system bleeds.

### 2.4 Statistical analysis

Qualitative variables were expressed as numbers and percentages while quantitative variables were expressed as means and standard deviations and/or medians and interquartile ranges (IQR). We performed univariate and multivariate analyses using R statistical software package [9]. We imputed the missing data using the random forest algorithm implemented in MissForest R package [10]. We used the univariate analyses, which included the chi-square test, two-sample t-tests and Mann-Whitney *U* test, to assess the degree of statistical significance between the risk factors and the three selected outcomes, described above. We set a p-value equal or less than 0.1 as a threshold for selecting the risk factors for the subsequent multivariate analyses. Because our sample was small (n = 122) we used a multivariable exact logistic regression model. Our multivariate analyses include three independent logistic regression models for each of the selected outcomes. We used a backward elimination approach and selected the final models based on the largest pseudo R^2^ as well as the smallest Akaike information criterion. Confounding by the demographic characteristics of the patients was assessed using the 10 % threshold change in the regression coefficient approach. Finally, we evaluated how well the final models fit the data using the Hosmer – Lemeshow goodness-of-fit statistic.

The study was compliant as per the STROCSS criteria [11] and is registered with Research Registry registration ID - researchregistry6817 which is accessible using this link https://www.researchregistry.com/register-now#user-researchregistry/registerresearchdetails/609eaae8d64e07001bf6eb34/

## 3 Results

We were consulted to manage bleeding episodes in 122 COVID-19 patients. Overall mortality was 59 % (n = 72). World health organization grades 2 and 3 were the most common bleeding grades in the series. The distribution of WHO bleeding grades were; WHO 0 (n = 6), WHO 1 (n = 19), WHO 2 (n = 56), WHO 3 (n = 40), WHO 4 (n = 1). Overall, the prevalence of invasive intervention was 11.5 % (14 patients), all were successful in resolving the bleeding event.

### 3.1 Dose of anticoagulation

Overall, 79 patients (67.7 %) were on therapeutic anticoagulation, 15 patients (12.3 %) were on intermediate dose, 22 patients (18 %) were on prophylactic dose and 6 patients (5 %) were on none. The distribution of anticoagulation doses per bleeding event and the need for intervention are summarized in Table 1. For details regarding individual bleeding events and interventions used to investigate and control the bleeding see supplementary materials and Table 1.

**Table 1:** Type of bleeding events, anticoagulation dose distribution, the need for intervention and cause of 30 Days mortality.

Type of bleeding	n	Dose of anticoagulation	Invasive Intervention	Mortality within 30 days
Retroperitoneal Bleed	9	Therapeutic dose n = 5 (55.5 %) Intermediate n = 0 Prophylactic dose n = 3 (33.3 %) None n = 1 (11.1 %)	Two patients required angioembolization, both were on prophylactic dose	2 patients died from COVID-19 sepsis
Intraperitoneal Bleed	2	Therapeutic n = 2 (100 %) Intermediate n = 0 Prophylactic n = 0 None n = 0	Two patients required angioembolization, both were on therapeutic dose	One patient died from COVID-19 sepsis
Abdominal Wall Hematoma	5	Therapeutic dose n = 4 (80 %) Intermediate dose n = 1 (20 %) Prophylactic n = 0 None n = 0	One patient required angioembolization, who was on therapeutic dose	One patient died from COVID-19 sepsis
Hematuria	12	Therapeutic dose n = 5 (41.6 %) Intermediate dose n = 3 (25 %) Prophylactic dose n = 4 (33.3 %) None n = 0	None	Two patients died from COVID-19 sepsis
Brain Bleed	7	Therapeutic n = 5 (71.5 %) Intermediate n = 0 Prophylactic n = 0 None n = 2 (28.5 %)	None	1 patient died from COVID-19 sepsis 4 patient died as direct consequence of the bleeding event
Nasopharyngeal Bleed	25	Therapeutic dose = 18 (72 %) Intermediate dose n = 3 (12 %) Prophylactic dose n = 4 (16 %) None n = 0	None	15 patients died from COVID-19 sepsis
Gastrointestinal bleeds	55	Therapeutic dose n = 34 (62 %) Intermediate dose n = 6 (11 %) Prophylactic dose n = 11 (20 %) None n = 4 (7.2 %)	- one patient required epinephrine injection with APC (on therapeutic dose) - Three patients required APC (all therapeutic dose) o Pyloric ulcer o Duodenal ulcer o Cecal ulcer - Three patients Gold Probe for duodenal ulcers (two patients on intermediate dose and one on therapeutic dose) - One Patient hemoclips for duodenal ulcer (on therapeutic dose)	38 patients; - 2 patients following unclear source of bleeding and died while still bleeding - The rest of patients died from COVID 19 sepsis

**Table 2:** Patients Demographics Baseline Characteristics. Significant p-values are boldfaced.

Characteristic	Mortality	P- value	Resolution of bleeding event	P-value	Having a GI bleed	P- value
Age (mean ± SD^1^)	60.26 (±1.82)	0.25	59.07 (±1.52)	0.77	60.52(±2.21)	0.30
Gender (Male)	58(80.56 %)	0.01*	78 (72.22 %)	0.00*	44 (80 %)	0.08
Length of Stay (mean ± SD)	25.30 (1.90)	0.02*	28.59 (±1.63)	0.37	27.35(±2.36)	0.65
Source of infection (community acquired)	68 (94.44)	0.09	106 (98.15 %)	0.01*	52 (94.55 %)	0.22
Location (ICU)	64(88.89 %)	0.00*	84 (77.78 %)	0.18	36 (65.45 %)	0.00*
*Clinical features upon admission*						
Hemoglobin on admission (g/L; mean ± SD)	110.68 (±3.20)	0.61	111.89 (±2.52)	0.85	111.94 (±3.63)	0.93
						
hypertension	44 (61.11 %)	0.09	58 (53.70 %)	0.45	31 (56.36 %)	0.77
Asthma/COPD***	9 (12.5 %)	0.67	11 (10.19 %)	0.21	4 (7.27 %)	0.18
Diabetes		0.96		0.23		0.27
None	37 (51.39 %)		58 (53.70 %)		39 (58.21 %)	
Uncomplicated	17 (23.61 %)		26 (24.07 %)		15 (22.39 %)	
End organ damage	18 (25 %)		24 (22.22 %)		13 (19.40 %)	
History of Myocardial infarction	15 (20.83 %)	0.50	19 (17.59 %)	0.32	11 (20 %)	0.76
Patient on ACEI/ARB^2^ medications	14 (19.44 %)	0.54	25 (23.15 %)	0.17	9 (16.36 %)	0.22
Patient on antiplatelets therapy before admission		0.36		0.53		0.71
yes	18 (25 %)		23(21.30 %)		13 (23.64 %)	
no	54 (75 %)		85(78.70 %)		42 (76.36 %)	
*Inpatient therapies and Complications developed during patients' admissions*						
Use of proton pump inhibitors		0.04*		0.06		0.00*
None	3 (4.17 %)		7 (6.48 %)		6 (10.91 %)	
Prophylactic	53 (73.61 %)		87(80.56 %)		33 (60 %)	
Therapeutic	16 (22.22 %)		14(12.96 %)		16 (29.09 %)	
Vasopressor	45 (62.50 %)	0.04*	57 (52.78 %)	0.18	27 (49.09 %)	0.24
Inotropes	23 (31.94 %)	0.00*	22 (20.37 %)	0.92	22 (40 %)	0.00*
Patient on antiplatelet therapy during admission		0.21		0.26		0.97
Yes	20 (27.78 %)		24(22.22 %)		13 (23.64 %)	
no	52 (72.22 %)		84(77.78 %)		42 (76.36 %)	
Anticoagulation dose on admission		0.00*		0.50		0.65
None	2 (2.78 %)		6 (5.56 %)		4 (7.27 %)	
Prophylactic	5 (6.94 %)		21(19.44 %)		11 (20 %)	
Intermediate	8 (11.1 %)		13(12.04 %)		6 (10.91 %)	
Full	57 (79.17 %)		68(62.96 %)		34 (61.82 %)	
Type of anticoagulation used		0.09		0.16		0.06
Enoxoparin	34 (48.57 %)		59(57.84 %)		28 (54.90 %)	
Fondoparinux	4 (5.71 %)		4(3.92 %)		4 (7.84 %)	
Unfractionated heparin	32 (45.71 %)		39(38.24 %)		19 (37.25 %)	
Inhaled steroid	11 (15.28 %)	0.39	16 (14.81)	0.12	11 (20 %)	0.04*
Systemic steroid (dose in mg)	748.33 (±98.60)	0.10	665.54 (±67.96)	0.85	676.87(±110.99)	0.82
systemic steroid		0.00*		0.77		0.54
yes	56 (77.78 %)		73 (67.59 %)		39 (70.91 %)	
no	16 (22.22 %)		35 (32.41 %)		16 (20.09 %)	
Oxygen therapy		0.00*		0.36		0.00*
Nasal	1 (1.39 %)		9(8.74 %)		8 (14.55 %)	
Mask	0		5(4.85 %)		0	
Invasive	71 (98.61 %)		89(86.41 %)		47 (85.45 %)	
						
ARDS		0.00*		0.00*		0.21
Normal to mild	6 (8.33 %)		24(23.30 %)		14 (25.45 %)	
Moderate to severe	66 (91.67 %)		79(76.70 %)		41 (74.55 %)	
Cardiac injury	22 (30.56 %)	0.00*	24(22.22 %)	0.49	23 (41.82 %)	0.00*
Liver injury	11 (15.28 %)	0.04*	11(10.19 %)	0.64	12 (21.82 %)	0.00*
Acute kidney injury	56 (77.78 %)	0.01*	75(69.44 %)	0.88	40 (72.73)	0.50
Renal replacement therapy	28 (38.89 %)	0.08	35(32.41 %)	0.80	25 (45.45 %)	0.00*
GCS less than 15	67 (93.06 %)	0.00*	80(74.07 %)	0.12	46 (83.64 %)	0.08
Respiratory rate more than 22	58 (80.56 %)	0.11	83 (76.85 %)	0.30	47 (85.45 %)	0.02*
Systolic blood pressure less than 100	38 (52.78 %)	0.00*	42 (38.89 %)	0.45	39 (70.91 %)	0.00*
Sepsis	57 (79.17 %)	0.00*	69 (63.89 %)	0.97	47 (85.45 %)	0.00*
qSOFA score		0.00*		0.33		0.00*
0	0		12 (11.11 %)		5 (9.09 %)	
1	13 (18.06 %)		22 (20.37 %)		5 (9.09 %)	
2	28 (38.89 %)		40 (37.04 %)		9 (16.36 %)	
3	31 (43.06 %)		34 (31.48 %)		36 (65.45 %)	
CCI score		0.61		0.20		0.28
0	13 (26 %)		21 (19.44 %)		10 (18.18 %)	
1	6 (12 %)		14 (12.96 %)		6 (10.91 %)	
2	7 (14 %)		20 (18.52 %)		7 (12.73 %)	
3	8 (16 %)		19 (17.59 %)		12 (21.82 %)	
4	5 (10 %)		11 (10.19 %)		5 (9.09 %)	
5	5 (10 %)		7 (6.48 %)		5 (9.09 %)	
6	2 (4 %)		7 (6.48 %)		3 (5.45 %)	
7	1(2 %)		2 (1.85 %)		1 (1.82 %)	
8	2 (4 %)		4 (3.70 %)		4 (7.27 %)	
9	1 (2 %)		3 (2.78 %)		2 (3.64 %)	
WHO grade		0.05		0.00*		0.00*
0	0		6 (5.56 %)		6 (10.91 %)	
1	8 (16 %)		13 (12.04 %)		3 (5.45 %)	
2	26 (52 %)		51 (47.22 %)		20 (36.36 %)	
3	16 (32 %)		38 (35.19 %)		26 (47.27 %)	
4	0		0		0	
*At consultation Symptoms, laboratory values, and interventions*						
Hematemesis	10 (13.89 %)	0.76	14(12.96 %)	0.89	14 (74.55 %)	0.00*
Melena	26 (36.11 %)	0.09	28 (25.93 %)	000*	29 (52.73 %)	0.00*
Occult bleed	11 (15.28 %)	0.04*	13 (12.04 %)	0.17	11 (20 %)	0.00*
Hemoglobin on consult day (g/L; mean ± SD)	77.58 (±2.26)	0.02*	81.81 (±2.14)	0.39	75.30 (±3.25)	0.00*
White blood cell (10^9^/L mean ± SD)	17.38 (±1.20)	0.00*	15.63 (±0.87)	0.92	16.49 (±1.39)	0.31
Neutrophil (10^9^/L; mean ± SD)	15.88 (±1.40)	0.08	14.25 (±1.19)	0.81	13.18 (±1.13)	0.32
Lymphocytes (10^9^/L; mean ± SD)	1.85 (±0.67)	0.87	1.44 (±1.44)	0.01*	1.18 (±0.11)	0.17
Hematocrit (L/L; mean ± SD)	0.26 (±0.01)	0.22	0.87 (±0.59)	0.70	0.26 (±0.01)	0.36
Platelet (10^9^/L; mean ± SD)	199.41(±14.48)	0.00*	237.01 (±13.05)	0.20	219.87 (±17.03)	0.41
Prothrombin time (seconds; mean ± SD)	16.81 (±0.58)	0.05	16.03 (±0.39)	0.16	16.17 (±0.38)	0.91
Activated partial thromboplastin time (seconds; mean ± SD)	48.15 (±2.63)	0.05	44.77 (±2.03)	0.44	45.98 (±3.39)	0.72
International normalized ratio	1.23 (±0.03)	0.01*	1.17 (±0.01)	0.00*	1.19 (±0.02)	0.66
D dimer (ng/mL; mean ± SD)	3421.37 (±234.11)	0.03*	3030.11 (±203.31)	0.34	3062.92 (±311.61)	0.88
Fibrinogen (g/L; mean ± SD)	5.24 (±0.18)	0.12	5.38 (±0.12)	0.51	5.36 (±0.17)	0.74
Estimated glomerular filtration rate eGFR (mL/mins/1.73m^2^; mean ± SD)	42.31 (±3.81)	0.00*	50.48 (±3.54)	0.31	45.36 (±4.95)	0.27
Urea (mmol/L)	26.19 (±1.74)	0.00*	22.37 (±1.42)	0.33	26.17 (±2.27)	0.02*
Creatinine (μmol/L; mean ± SD)	255.44 (±24.98)	0.02*	214.38 (±18.14)	0.21	246.34 (±27.41)	0.21
CRP (mg/L; mean ± SD)	173.69 (±12.01)	0.00*	144.71 (±9.76)	0.01*	145.25 (±15.22)	0.44
Procalcitonin (ng/mL; mean ± SD)	11.91 (±3.16)	0.11	7.20 (±1.11)	0.00*	5.44 (±0.73)	0.06
Noninvasive intervention	60 (83.33 %)	0.69	92 (85.19 %)	0.52	41 (74.55 %)	0.00*
Invasive intervention	12 (16.67 %)	0.92	17 (15.74 %)	0.59	14 (25.45 %)	0.01*
Hemostatic intervention	6 (8.33 %)	0.31	13 (12.04 %)	0.17	8 (14.55 %)	0.20

1. SD: Standard deviation.

2. ARB/ACE: angiotensin receptors blocker/angiotensin converting enzyme inhibitor.

**Table 3:** Multivariate Logistic Regression Model for Potential Risk Factors Associated with Inpatient Mortality. Significant p-values are boldfaced.

Risk factor	Odds ratio	95 % confidence interval	P-value
Length of Stay	0.95	0.92–0.98	**0.00**
Anticoagulation dose on admission			
Theraputic	Reference	-	-
Prophylactic	0.07	0.02–0.28	**0.03**
Intermediate	0.36	0.09–1.34	0.13
None	0.10	0.01–0.86	**< 0.00**
Occult bleed			
No	Reference	-	-
Yes	15.0	1.97–29.1	**0.01**
White blood cell	1.08	1.02–1.15	**0.01**
Platelet	0.98	0.97–0.99	**0.00**

Hosmer – Lemeshow goodness-of-fit p-value = 0.612.

**Table 4:** Multivariate Logistic Regression Model for Potential Risk Factors Associated With Resolved Bleeding Events. Significant p-values are boldfaced.

Risk factor	Odds ratio	95 % confidence interval	P-value
Use of proton pump inhibitors			
None	Reference	-	-
Prophylactic	13.04	3.2–136.94	**0.03**
Therapeutic	1.24	0.13–14.84	0.86
Melena			
No	Reference	-	-
Yes	0.03	0.01–0.18	**< 0.00**
Melena			
GI bleed	-	-	-
No GI bleed	0.03	0.01–0.26	**0.00**
CRP	0.98	0.97–0.99	**0.00**

Hosmer – Lemeshow goodness-of-fit p-value = 0.130.

**Table 5:** Multivariate Logistic Regression Model for Potential Risk Factors Associated with the Type of Bleeding Event (having a GI bleed). Significant p-values are boldfaced.

Risk factor	Odds ratio	95 % confidence interval	P-value
Inotropes			
No	Reference	-	-
Yes	7.33	1.03–55.28	0.05
Cardiac injury			
No	Reference	-	-
Yes	6.73	0.92–49.43	0.06
Liver injury			
No	Reference	-	-
Yes	74.08	4.18–132.08	**0.00**
Q-sofa score			
0	Reference	-	-
1	0.19	0.02–1.89	0.15
2	0.08	0.06–2.18	0.06
3	23.43	4.94–374.73	**0.02**
Hematemesis			
No	Reference	-	-
Yes	19.79	2.23–175.74	**0.00**
Occult bleed			
No	Reference	-	-
Yes	32.24	3.34–311.08	**0.00**

Hosmer – Lemeshow goodness-of-fit p-value = 0.99.

### 3.2 Univariate analysis - Table 2

#### 3.2.1 Mortality outcome

Male patients (80.56 %) were significantly more likely to die than female patients. The mean age for patients who died was 60 years old, they were significantly more likely to be admitted to intensive care unit (ICU) and had shorter hospital stay compared to patients who survived. Medical comorbidities at the time of admission did not have significant implication on the risk of mortality. These included having a history of hypertension requiring medications, asthma or chronic obstructive pulmonary disease (COPD), having complicated diabetes, remote history of myocardial infarction, or being on antiplatelets (single or dual) at the time of admission. Moreover, admission baseline hemoglobin level, before the onset of bleeding episode, did not affect the risk of mortality. The risk of death appears to increase in patient who were on PPI, vasopressors, inotropes, systemic steroids, and those who were on invasive ventilation and having severe ARDS.

The dose but not the type of anticoagulation at the time of admission was significantly associated with risk of death following the bleeding episode. Moreover, having a cardiac injury, liver injury, acute kidney injury, Glasgow coma scale (GCS) less than 15, systolic blood pressure less than 100 and sepsis and high qSOFA score were associated with higher risk of death. However, the CCI score and the WHO bleeding grade did not affect the risk of death.

We also found that having an occult source of bleeding rather than a specific symptoms and signs indicative of a source of bleeding was significantly associated with the risk of death. There was no relationship between the type of intervention (invasive, noninvasive or hemostatic) and the risk of death within 30 days.

Furthermore, we found having a GI bleeding event was significantly associated with the risk of death (P = 0.04). The prevalence of death following bleeding event was higher following a GI bleed compared to other causes of bleed, 52.7 % vs. 48.3 % respectively.

#### 3.2.2 Resolution of bleeding event outcome

We found that the dose of anticoagulation, being on systemic steroid, having severe ARDS did not affect bleeding event resolution. None of the complications the patients developed during admission affected bleeding resolution chance as well. Furthermore, we did not identify a significant relationship between any coagulation profile derangements and the ability to control bleeding episode. However, WHO bleeding grade, was significantly associated with bleeding resolution.

#### 3.2.3 Type of bleeding outcome

We found that having cardiac injury, liver injury, being on renal replacement therapy, having a respiratory rate over 22, being on invasive ventilation, having systolic blood pressure less that 100, and being septic to significantly influence the type of bleeding event. Also, the qSOFA and WHO bleeding grade but not CCI score significantly affected the type of bleeding event. We also found GI bleeds to be significantly associated with the need to use PPI and inotropes but not vasopressors.

We also found that the dose of anticoagulation, having severe ARDS, being on systemic steroids did not affect the type of bleeding event outcome. However, having an occult bleed appeared to be significantly associated with having a GI bleed. Furthermore, having high urea but not deranged coagulation profile was significantly associated with having a GI bleed. Moreover, there was a significant relationship between the type of intervention (invasive, noninvasive or hemostatic) and the type of bleeding event.

#### 3.2.4 Laboratory values association with mortality and bleeding resolution outcomes

We identified a significant association between admission hemoglobin level, white blood cell (WBC) count, platelet, international normalized ratio (INR), D-dimer level, e-glomerular filtration rate (eGFR) level, urea, creatinine, and C reactive protein (CRP) and the risk of death.

We also identified significant association between on admission CRP and Procalcitonin (PCT) levels and the risk of bleeding resolution.

### 3.3 Risk factors predictors of primary outcomes – multivariate logistic regression

#### 3.3.1 Mortality - Table 3

Patient who had longer hospital stay appeared to be less likely to die, odds ratio (OR) 0.95 (95 % CI, 0.92–0.98, p = 0.003). With regards to the dose of anticoagulation, we found that patients who were on intermediate dose, prophylactic dose or no anticoagulation were less likely to die than patients on therapeutic dose anticoagulation. This risk appears to be significant when prophylactic dose anticoagulation and no anticoagulation were compared to therapeutic dose, OR 0.07 (95%CI 0.02–0.028, p = 0.03) and OR 0.1 (95%CI 0.97–0.99, p < 0.00) respectively but not significant when compared intermediate dose was compared to therapeutic dose, OR 0.36 (95%CI 1.02–1.15, p = 0.13). Furthermore, having an occult bleeding appeared to be a significant predictor of risk of death, OR 15 (95 % CI 1.97–29.1, p = 0.01). Also, WBC and platelet levels appeared to independently affect risk of death.

#### 3.3.2 Resolution of bleeding event - Table 4

Patients who were on PPI were more likely to have resolution of bleeding event compared to patients who were not. Out of all GI symptoms and signs melena appeared to be significantly associated with lower odds of bleeding resolution, OR 0.03 (95%CI 0.01–0.18, p < 0.00). C-reactive protein appeared as well to be significantly associated with lower odd of bleeding resolution, OR 0.98 (95 % CI 0.97–0.99, p < 0.00).

#### 3.3.3 Type of bleeding event - Table 5

The risk of GI bleeding increased when patients were on inotropes (OR 7.33, 95%CI 1.03–55.28, p = 0.005), had cardiac injury (OR 6.73, 95%CI 0.92–49.43, p = 0.06), had liver injury (OR 74.08, 95%CI 4.18–132.08, p = 0.03), had qSOFA score of 3 (OR 23.43, 95%CI 4.94–374.73, p = 0.02), had hematemesis (OR 19.79, 95%CI 2.23–175.74 p = 0.00), and had occult bleed (OR 32.24, 95%CI 3.34–311.08, p = 0.00). The mortality variable had poor correlation with the type of bleeding event on multivariate analysis model and so it was removed from the model.

## 4 Discussion

Our study was the first to identify a significant association between the dose of anticoagulation in COVID-19 patients and the risk of death. Specifically, we found patients who were on prophylactic dose or no anticoagulation to have lower risk of death compared to patient on therapeutic anticoagulation by 7 %, and 10 % respectively. When therapeutic dose was compared to intermediate dose, there was no significant difference in the risk of death.

Due to the hypothesized hemostatic derangement observed with COVID-19 which causes a microthrombosis induced multiorgan failure and death [12] clinicians have been routinely prescribing intermediate and full therapeutic doses rather than prophylactic dose anticoagulation to prevent this presumed phenomenon. Helms et al. recently reported at least 40 % thrombotic complications in patients with COVID-19 [13]. Bao et al. [14] compared platelet and coagulation functions in patients with severe COVID-19 disease to patients with non-severe disease. They found the patients with severe disease had lower platelet function, more prolong bleeding time and higher incidence of DIC than non-severe group. Zhang et al. [15] found using both human and animal models that the low platelet activity found in severe COVID-19 patients was associated with increase platelet volume (mean platelet volume) and platelet hyperactivity. These in consequence were found to enhances platelet aggregation and activation of various coagulation factors resulting in increased risk of thrombosis. Franklin et al. [16] tested the efficiency of blood coagulation pathways using thromboelastography in 44 COVID-19 patients. They found that critically ill patients had higher d-dimer and slower clotting which were significantly associated with 50 % increase in risk of thromboembolic events. Similar findings were reported by Pavoni et al. [17] and Tang et [18]. al. has suggested mortality benefits with the use of anticoagulation in COVID-19 patients. However, all these studies suffer from small samples size and limited exploration of the known potential negative implications of higher doses of anticoagulation use. In our population we noticed that the majority of patients who had head and neck bleeds (brain and NPA) ended up dying (see supplemental materials for details). Patients with brain bleeds who died were deemed inoperable (because by the time it was diagnosed intervention seems too late in typical patient with limited physiological reserve in context of COVID-19 sepsis) and all of them were on therapeutic anticoagulation. Dogra et al. [19] reported 4.4 % of 755 patients diagnosed with COVID-19 were found to have ICH on concurrent neuroimaging, of whom the majority of these patients were on therapeutic anticoagulants. In our population, of all deaths in NPA bleeds all were on therapeutic and or intermediate dose anticoagulation except one. However, despite bleeding resolution in all NPA patients events, significant proportion of patients eventually expired. This might be because the progressive “lingering” COVID-19 associated platelet dysfunction and DIC rather than the acute bleeding event itself are the major contributors to the eventual death of patient with COVID-19 and so NPA bleeds should be considered red flags for aggressive persistent COVID-19 coagulopathy, multiorgan failure and eventual death. Thus, efforts should be focused on correction and optimization of COVID-19 sepsis therapies and anticoagulation should be administered with caution in head neck bleeds subpopulation. Our findings in the context of the available literature put into question the routine unopposed practice of prescribing “supra-prophylactic dose” anticoagulation to newly admitted COVID-19 patients and probably these doses should be prescribed in selective cases only.

Moreover, we found that the dose of anticoagulation did not influence the risk of bleeding resolution. This might suggest that regardless of the dose of anticoagulation patient is on, noninvasive interventions such as withholding anticoagulation following bleed event, rather than an invasive or hemostatic intervention are the major determinants of bleeding resolution, and so should always be considered as first and primary line of intervention. This approach will save valuable resources and spare health care professionals unnecessary exposure.

Samkari et al. [5] retrospective study of 400 admitted COVID-19 patients who were primarily receiving prophylactic dose of anticoagulation reported thrombocytopenia at initial presentation to be significant predictor of bleeding. In our study, we found, “chemical thrombocytopenia”, being on antiplatelets therapy (single, dual or even the novel ones), did not affect the risk of mortality, resolution of bleeding events nor the type of bleeding. This might indicate that sepsis induced platelet dysfunction and eventually shock rather than thrombocytopenia itself significantly interact with bleeding events variables.

Moreover, WHO grade, which is representative of the volume of blood lost and thus indirectly blood transfusion requirements, did not affect the risk of mortality in our population. The majority of the bleeding events encountered in our population were WHO 2 or 3 (78.6 %). This moderate degree of bleeding likely did not lead to hemodynamic instability and thus did not generate enough force to tip the patient toward shock when they were not or worsened an existing shock. Furthermore, we found newly developed, in hospital, medical comorbidities rather than pre-existing ones before admission to carry more weight on increasing the risk of mortality following a bleeding event. Charlson score (CCI), contains both pre-existing and newly developed medical comorbidities, and qSOFA score contains only acute ones. We found CCI score did not to affect the risk of mortality, but qSOFA score, acute cardiac injury, acute liver injury, acute kidney injury, in hospital GCS less than 15, in hospital systolic blood pressure less than 100, and sepsis, were significantly associated with increased risk of mortality.

In general, in acute GI bleeding events, endoscopy remains the first line intervention within 24 h of patient stabilization. However, with the era of COVID-19, the risk benefits equation got more complex by concerns for provider safety and a need to preserve personal protective equipment. Moreover, there are limited data on the diagnostic and therapeutic benefits of endoscopy in this cohort, leaving endoscopist with inadequate information and algorithms to guide their decision of when the risk of endoscopy outweigh the benefits. In our population the prevalence of instigating diagnostic or therapeutic endoscopic is relatively low. However, this does not appear to be unique to our center. Salerno et al. [20] looked at the impact of COVID-19 on urgent endoscopy in Italy. They reported a significant reduction in the number of urgent upper and lower GI endoscopy by 80 % and 55 % respectively. This reduction in endoscopy use was replicated in a Belgium study which reported 40 % reduction in upper GI bleeding events requiring endoscopy [21]. Salerno et al. also reported that the significant reduction in endoscopy use was associated with increase in diagnostic yield by over 10 % in the upper GI endoscopy group. This correlate well with our findings. Where even though our use of endoscopy to investigate our GI bleeding events was relatively low, as endoscopy was preserved for patients with clinically significant GI bleed and those who failed conservative therapy, our diagnostic yield was relatively high for both upper and lower GI endoscopy, 88.8 % and 60 % respectively. Martin et al. [22] conducted a match case control study of 41 patients with COVID-19 who had bleeding events (31 upper GI and 10 lower GI bleeds) compared to 82 COVID-19 patients who did not have GI bleeds. They found no difference in presenting symptoms and signs, no difference in severity of COVID-19 manifestations, and no difference in anticoagulation use. They reported most common cause of upper GI bleed was duodenal ulcer (80 %), ours was 55.5 % duodenal & gastric ulcers. For lower GI bleeding event they reported rectal tube insertions to be the most common cause (60 %), our hospital does not use rectal tubes routinely. In their study, hemostatic interventions where successful in all cases who required intervention (n = 7), with no immediate postprocedural complications happened, and no interventional radiology or surgical procedures were required. We had similar experience with 100 % successful interventions for both non-GI and GI bleeds for all hemostatic interventions attempts. On multivariate analysis they found having a previous history of upper GI bleed to be the only predictor of upper GI bleeding. We found being on inotropes, having liver injury, high qSOFA score, having hematemesis, and occult bleeding to be significant predictor of having a GI bleed. Furthermore, they found trends toward higher risk of upper and lower bleeding events with being on anticoagulation, but this was not statically significant. We as well did not find a significant relationship between the dose of anticoagulation and the risk of having a GI bleed over other causes of bleeding. Similar to Martin et al. the majority of our study bleeding events ultimately had cessation of bleeding without the need for hemostatic intervention. Furthermore, our GI bleeding population had high mortality rate (69 %) despite bleeding resolution in the majority of cases, indicating patient are likely dying from other causes rather than delay in GI bleeding recognition or treatment. Based on all that, in COVID-19 era it appears to be safe to delay instigating endoscopic an intervention for GI bleeds, contrary to most guidelines which recommends early intervention for bleeding events. This will help alleviate concerns toward patient's respiratory status or illness severity, provider safety, PPE conservation, and the preservation of ventilators and avoiding procedural related intubations.

Finally, we found having a GI bleed was significantly associated with increased risk of mortality (p = 0.04), despite bleeding event resolution in the majority of cases. We also found having an occult bleed was associated with 15 times increased risk of death. This “again” suggests that slow non profound bleeding as a consequence of COVID-19 coagulopathy might be a more significant contributor to increased risk of mortality rather an acute bleeding episode which is normally associated with hard signs of bleeding such as hematemesis and melena. Based on that, efforts probably should be focused on correction of coagulopathy and sepsis rather than none targeted invasive GI interventions in patient with COVID-19, since most of these patients do not have identifiable source of bleeding amendable to invasive hemostatic intervention.

### 4.1 Strengths & limitations

Principle strength of the study is its inclusion of both detailed narrative and quantitative statistical analyses of one of the largest available series of bleeding COVID-19 patients’ data in a research field that lacks studies with enough sample size to make meaningful conclusions. Furthermore, this study captured the majority of bleeding COVID-19 patients who were on anticoagulation in Kuwait, since the tertiary care center used to collect patients data was the only major dedicated COVID-19 admitting center in Kuwait during the study period.

One major limitation of the present study was that it was derived from a single institutional cross-sectional study with inherent selection and information bias, hence generalizability of the findings to larger populations might not be representative. Further, our study had a limited sample size which led to the inflation of the ORs 95 % CI, rendering them notably less precise. However, our inferences were based on all available data on rare outcomes that were collected within a short time during the current pandemic. Therefore, future studies should be focused on collecting more data to additionally validate our results.

## 5 Conclusions

As COVID-19 pandemic evolves, we are being confronted with unique challenges, particularly understanding the bleeding sequala of this novel virus. With the increasing use of supra-prophylactic doses of anticoagulation in this subpopulation the incidence of bleeding events, will be on the increase. We are the first to identify a significant association between the dose of anticoagulation and risk of mortality. The previously unchallenged recommendation to prescribe therapeutic and or intermediate doses of anticoagulation to all newly admitted patients with COVID-19 should be revisited and more individualized approach to prescription should be the norm. The best approach to manage COVID-19 bleeding patients is to prioritize therapies that manage sepsis induce coagulopathy and shock over other approaches.

It appears that the majority of bleeding events in COVID-19 patients resolve with noninvasive interventions and when hemostatic interventions were necessary it had high success rate, despite the delay. This means that conservative management at the time of consultation seems to be a reasonable initial approach managing these complex cases, as most cases will resolve without the need for intervention. This alleviates concerns regarding provider safety, and the need to preserve personal protective equipment without jeopardizing patient safety and outcomes.

With regards to GI bleeds, endoscopic intervention should be limited to patients with hard signs of GI bleeds, such as hematemesis or melena. In patients with occult bleed efforts might be better geared toward optimizing therapies that manage COVID-19 sepsis induced coagulopathy, DIC and shock rather than none target low yield endoscopic interventions. The same principle applies to patients with NPA bleeds, as it should be considered a sign for lingering progressive COVID-19 sepsis and coagulopathy and thus efforts should be focused on correction and optimization of COVID-19 sepsis therapies.

Further clinical research into the pathogenesis of COVID-19 and its complications is needed as till now no clear validated treatment guidelines for the management of the disease and its complications including thromboembolic complications are available.

## Ethical approval

The Ministry of Health of Kuwait which is the government branch at the state of Kuwait which manage Jabir AL-Ahmad Hospital (our study centre) approved the study. Ethical approval number. 1402/2020.

## Funding statement

This work was supported by 10.13039/501100003286Kuwait Foundation for the Advancement of Science (KAFAS), a government non-profit organization (grant number: CRONOA PROP 56).

## Author contributions

All authors read and approved the manuscript

AA contributed to study concept, design, data collection, analysis, draft preparation and final submission YA contributed to study concept, design, data collection, analysis, draft preparation and final submission KA contributed to study concept, design, data collection, analysis, draft preparation and final submission EL contributed to study concept, design, data collection, analysis, draft preparation and final submission MA contributed to study concept, design, data collection, analysis, draft preparation and final submission AL contributed to study data collection, analysis, draft preparation and final submission MA contributed to study data collection, analysis, draft preparation and final submission MJ contributed to study concept, design, draft preparation and final submission SA contributed to study concept, design, draft preparation and final submission.

## Registration of research studies

1. Name of the registry: researchregistery
2. Unique Identifying number or registration ID: researchregistry6817
3. Hyperlink to your specific registration (must be publicly accessible and will be checked): https://www.researchregistry.com/register-now#user-researchregistry/?view_26_sort=field_21|desc

## Consent

Since the this is a retrospective review of prospectively maintained database, patient consent was not required to get ethical approval. To ensure patient confidentiality patient identification data were anonymized.

## Guarantor

Dr. Ahmed ALkhamis.

## Provenance and peer review

Not commissioned, externally peer-reviewed.

## Declaration of competing interest

Nothing to disclose.
