# Supplementary material for: Prevalence, predictors and outcomes of bleeding events in patients with COVID-19 infection on anticoagulation: Retrospective cohort study
Source: Ann Med Surg (Lond). 2021 Jul 21;68:102567. doi: 10.1016/j.amsu.2021.102567 (PMC8294630; doi:10.1016/j.amsu.2021.102567)
Supplement: Multimedia component 1 [file mmc1.docx]

**Narrative descriptive review of both Gastrointestinal (GI) and none GI bleeding events**

GI BLEEDING EVENTS

Out of 55 patients who had signs of GI bleed at initial presentation 47 patients were managed with full dose proton pump inhibitors (PPI) and active observation, of whom 6 failed this approach and required endoscopic intervention, five EGD and 1 colonoscopy.

Of the 19 patients who had endoscopic procedures 18 EGDs and 5 colonoscopies were performed. Nine patients (16.3%) required an endoscopic hemostatic intervention to control the bleeding, all were successful. Eight patients required upper GI hemostatic intervention and 1 lower GI hemostatic intervention. The upper GI interventions included gold probe, epinephrine injection, APC and hemoclips applications for bleeding ulcers. Bleeding resolved for all cases. Etiology for bleeding events were duodenal & gastric ulcers in 10 patients (55.5%), esophagitis and Roux-en-Y anastomosis ulcer in 1 patient, and gastritis in 5 patients (27.7%). Three patients died within 30 days of consultation. The only lower GI endoscopic intervention was for large colonic ulcer in the cecum which was treated with APC. Bleeding resolved but patient later died from sepsis. Other colonoscopies done without interventions were for; small rectal ulcer with piles for 1 patient, ischemic colitis for 1 patient, piles for 1 patient, and one colonoscopy was essentially normal.

Overall, of 55 patients with signs of GI bleed, 38 (69.1%) expired within 30 days of consultation. Two patients had unclear source of bleeding and died while still bleeding, both were on Plavix and therapeutic anticoagulation at the time of consultation. The rest died after resolution of the bleeding episode from COVID-19 complications.

NON-GI BLEEDING EVENT

*a) Retroperitoneal, intraperitoneal and abdominal wall hematoma*

We identified 9 cases of retroperitoneal bleeding (7.3%). Three patients were on prophylactic anticoagulation, five were on therapeutic dose and one was on none. Only one patient was on antiplatelet (aspirin) as well as anticoagulation. Two patients (both were on prophylactic dose) required angioembolization to control the bleeding and both were resolved. The rest resolved with no intervention. Of the 7 patients who did not require interventions 3 died within 30 days of consultation from sepsis.

We identified 2 intraperitoneal bleeding. Both patients were on therapeutic dose anticoagulation and both required angioembolization to successfully control the bleeding. However, one of the two patients died later within 30 days from sepsis.

Five patients with abdominal wall hematomas were encountered. One patient was on intermediate dose and 4 on therapeutic dose. All bleeding resolved with noninvasive interventions except one which requited angioembolization to control the bleeding. Of all patients with abdominal wall bleeding, one died within 30 days after the consultation from COVID-19 multiorgan failure.

*b) Hematuria*

Twelve patients had hematuria. Four patients were on prophylactic dose, five on full dose and three on intermediate dose anticoagulation. All bleeding resolved without the need for invasive intervention. Two patients died within 30 days.

*c) Brain bleeds*

Seven patients had cerebral bleeding events. Two patients were not on anticoagulation; one patient developed subarachnoid hemorrhage which had stabilized without intervention, however, patient later died from sepsis and the other patient survived the bleeding event. Five patients were on therapeutic anticoagulation; 4 patients expired; one as a direct consequence from intracerebral bleeding (deemed inoperable), one following combined spontaneous subdural and epidural hematomas (deemed inoperable), and 2 had hemorrhagic infarcts.

*d) Nasopharyngeal bleeding*

We identified 25 patients who had NPA bleeding. Diagnoses ranged from epistaxis, oral bleeding and tracheal site bleeding. Interventions, which were all noninvasive, included holding the anticoagulation till bleeding resolved, nasal packing and administering vitamin K. All bleeding resolved with these techniques. Fifteen patients died within 30 days from sepsis. Of all NPA consultations, four patients were on prophylactic dose, 3 on intermediate dose, and 18 on therapeutic dose anticoagulation. Of patients who died, all were on therapeutic dose anticoagulation except two, one was on intermediate dose, and the other was on none.
